# Design and Fabrication of a Magnetic Actuator for Torque and Force Control Estimated by the ANN/SA Algorithm

**DOI:** 10.3390/mi13020327

**Published:** 2022-02-19

**Authors:** Pooriya Kazemzadeh Heris, Mir Behrad Khamesee

**Affiliations:** Department of Mechanical and Mechatronics Engineering, University of Waterloo, Waterloo, ON N2L 3G1, Canada; pkazemza@uwaterloo.ca

**Keywords:** electromagnetism, magnetic manipulator, magnetic actuator, deep learning, ANN, ANN/SA

## Abstract

Magnetic manipulation has the potential to recast the medical field both from an operational and drug delivery point of view as it can provide wireless controlled navigation over surgical devices and drug containers inside a human body. The presented system in this research implements a unique eight-coil configuration, where each coil is designed based on the characterization of the working space, generated force on a milliscale robot, and Fabry factor. A cylindrical iron-core coil with inner and outer diameters and length of 20.5, 66, and 124 mm is the optimized coil. Traditionally, FEM results are adopted from simulation and implemented into the motion logic; however, simulated values are associated with errors; 17% in this study. Instead of regularizing FEM results, for the first time, artificial intelligence has been used to approximate the actual values for manipulation purposes. Regression models for Artificial Neural Network (ANN) and a hybrid method called Artificial Neural Network with Simulated Annealing (ANN/SA) have been created. ANN/SA has shown outstanding performance with an average *R*^2^, and a root mean square error of 0.9871 and 0.0153, respectively. Implementation of the regression model into the manipulation logic has provided a motion with 13 μm of accuracy.

## 1. Introduction

Electromagnetism principles and magnetic actuators have been implemented in various applications such as metal forming, where a solenoid with 15 turns generates a magnetic force to push a light metal in order to form a cellphone case [1], real-time production of magnetic materials with the 3D printer in additive manufacturing is feasible by aligning the particles with the magnetic field [2]. In addition, magnetic levitation can be used to design a stable suspension for metal additive manufacturing, which results in substrate elimination [3]. For biological purposes, the electroless plating technique has been used to make magnetic microparts that can rotate at high speed using a magnetic actuator for functional lab-on-a-chip devices [4]. Microfluidics has also utilized magnetically actuated discs to control the flow, mixing, reaction, and separation of fluid [5]. Furthermore, the conversion of kinetic energy of human movement into electrical power is possible using a coil-spring system [6]. The density of dense non-magnetic materials, such as glass, is also measurable, by analyzing the strength of the levitation of a single iron-core coil (electromagnet) [7]. In another study, the variation of the eddy current produced by a coil is measured to detect the place and size of different defects [8]. In addition to the eddy current, electromagnetic impedance and magnetic field values in one direction are also utilized to develop various detection sensors, where one can detect a different contaminant in hydraulic oil [9] and measure the size and position of a contaminant during the casting process [10], respectively.

Magnetic actuators are also implemented in micro-electro-mechanical systems (MEMSs) such as micropumps [11], stable grasping [12], pick-and-place system [13], small force sensing [14], contactless delivery [15], and microsurgery [16]. Microsurgery can provide a minimally invasive surgery environment and offer benefits such as lower infection risk, fewer medical complications, and faster rehabilitation [17,18]. Bioengineering combined with the small-scale (millimeter and sub-millimeter) wireless robots manipulated via the magnetic field can potentially modernize the medical field [19] by replacing the current devices with safer alternatives that enable surgeons to access fragile organs such as the eye, heart, and brain to perform more precise and less invasive operations. It can also be utilized to navigate capsules inside the body for targeted delivery purposes and approach part of the body that was not safely accessible before [20].

Octomag is a magnetic actuator developed to enhance retinal surgery by eliminating irreversible damage to the eye through the control constraints that could even respond appropriately to unpredicted issues such as patient movement. This device contains eight electromagnets to perform a 5 degree of freedom (5 DOF), transition, and orientation, via magnetic force and torque control [21]. Octomag utilizes a linear relationship of fields generated by an individual coil to calculate the total magnetic field using the FEM approach. Then, calculated magnetic field values are calibrated with a few known values measured from the actual coil in order to form the control logic.

Yuan et al. have designed an eight metal-core coil actuator that adopted the same concept as the Octomag to navigate a capsule inside the stomach [22]. Minimag was also motivated by OctoMag, enabling 5 DOF inside a spherical working space surrounded by eight iron-core coils. This system also followed the same approach of calibrating the FEM simulations to develop its control logic [23].

Catheter positioning [24,25] is another main advantage of magnetic manipulators; the catheter guidance control and imaging (CGCI) systems use eight giant coils and have been implemented at an industrial size [26]. Moreover, Bigmag has used six moving coils to navigate a catheter [27].

**Figure 1:**
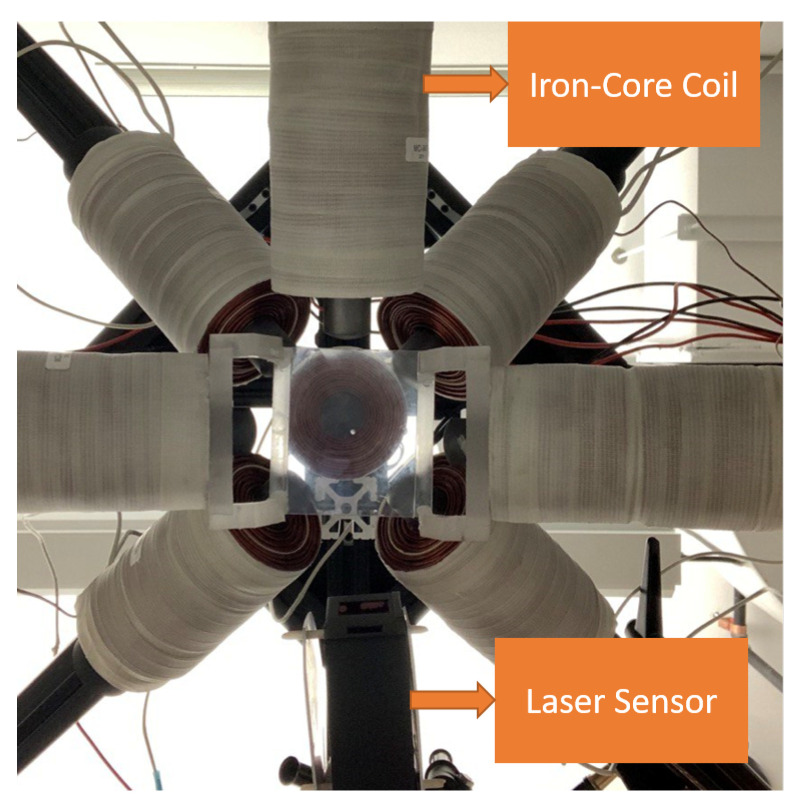
Magnetic manipulator consist of eight iron-core coils.

Figure 1 indicates the developed system utilized in this study to manipulate a disk magnet with a diameter and thickness of 0.1 and 0.0625 inches, respectively. The manipulator contains eight coils in an open-asymmetric configuration that enables wider access to the working space than the mentioned systems and does not compromise torque-force controllability within the workspace [28]. This configuration also follows the square antiprism of Thomson’s method [29] and can be modeled using the superposition theorem. This project assumes that the changes in a magnetic field are infinitely small; hence, Maxwell laws are applicable. Moreover, due to the high magnetic permeability of the pure iron core, the inserted core discharges almost instantly after getting magnetized; therefore, the slight non-linearity can be neglected, meaning that the superposition is still valid.

## 2. Method

### 2.1. Coil Design

**Figure 2:**
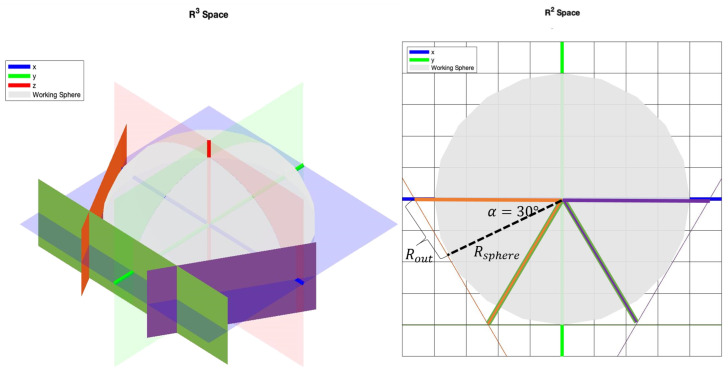
Working space and outer diameter of the coils.

As indicated in Figure 2, the radius of the spherical working space is correlated to the outer diameter of the coils. The surface area of coils is represented with planes, where the largest coil diameter is located at the intersection of the planes. The relationship between the largest outer radius of the coils, $R_{out}$, and spherical working space, $R_{sphere}$, in the open-asymmetric configuration, can be modeled as:

(1) $$R_{out}=\tan\left(\frac{\pi }{6}\right)\times R_{sphere}.$$

**Figure 3:**
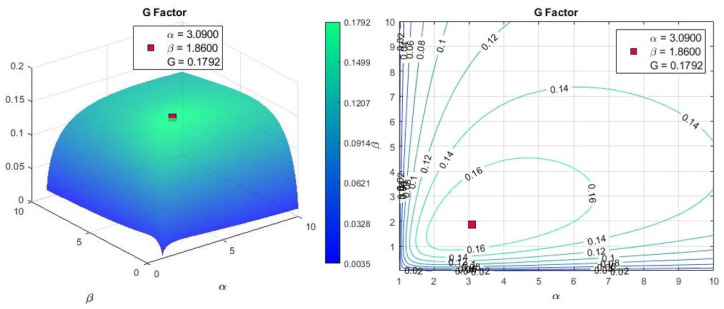
Ideal Fabry factor values for minimizing heat dissipation.

By equating the spherical radius to 60 mm, the outer diameter of the coil should be less than 69.2820 mm. The selection of the outer diameter leads to using the Fabry factor, *G*, to measure the coils’ inner radius and height.

(2) $$G=\frac{1}{5}\sqrt{\frac{2\pi \beta }{\alpha^{2}-1}}\ln\frac{\alpha +\sqrt{\alpha^{2}+\beta^{2}}}{1+\sqrt{1+\beta^{2}}}$$

$$\alpha =\frac{R_{out}}{R_{in}}$$

$$\beta =\frac{l}{2R_{in}}$$

where $R_{in}$ and *l* are the inner radius and length of the coil, respectively. The optimum values for these factors are shown in Figure 3.

*G* factor parameterizes the coil’s dimension, generating the least heat dissipation to produce a magnetic field. In this study, the G factor has been combined with the force generated on a 1-${\mathrm{mm}}^{3}$ magnet located at the center of the working space. Therefore, the coil’s parameters that generate the most force value with minimum power consumption will be selected as the final coil.

### 2.2. Magnetic Force and Torque

The generated magnetic force and torque of a coil can be calculated using Maxwell Law:

(3) $$\vec{\nabla }\cdot \vec{B}=0$$

(4) $$\vec{\nabla }\times \vec{B}=\mu_{0}\vec{J}$$

where *J*, *B*, and $\mu_{0}$ are the current density, magnetic field, and magnetic permeability of the free space. The force (*F*) and torque (τ) acting on an object with the dipole moment of $\vec{m}$ is equal to:

(5) $$\vec{F}=\left(\vec{m}\cdot \vec{\nabla }\right)\vec{B}$$

(6) $$\vec{\tau }=\vec{m}\times \vec{B}.$$

Equations (5) and (6) can be represented in matrix form [30]:

(7) $$\vec{F}=\left[\begin{array}{ccccc} m_{x} & m_{y} & m_{z} & 0 & 0\\ 0 & m_{x} & 0 & m_{y} & m_{z}\\ -m_{z} & 0 & m_{x} & -m_{z} & m_{y} \end{array}\right]\left[\begin{array}{c} \frac{\delta B_{x}}{\delta x}\\ \frac{\delta B_{x}}{\delta y}\\ \frac{\delta B_{x}}{\delta z}\\ \frac{\delta B_{y}}{\delta y}\\ \frac{\delta B_{y}}{\delta z} \end{array}\right]=h\left(\vec{m}\right)g\left(\vec{\nabla }{\vec{B}}^{T}\right)$$

(8) $$\vec{\tau }=\left[\begin{array}{ccc} 0 & -m_{z} & m_{y}\\ m_{z} & 0 & -m_{x}\\ -m_{y} & m_{x} & 0 \end{array}\right]\left[\begin{array}{c} B_{x}\\ B_{y}\\ B_{z} \end{array}\right]=S\left(\vec{m}\right)\vec{B}$$

and (7) and (8) can be integrated to form wrench, *W*:

(9) $$\vec{W}=\left[\begin{array}{c} \vec{\tau }\\ \vec{F} \end{array}\right]=\left[\begin{array}{cc} S(\vec{m}) & O\\ O & h(\vec{m}) \end{array}\right]\left[\begin{array}{c} \vec{B}\\ g(\vec{\nabla }{\vec{B}}^{T}) \end{array}\right]$$

where *O* is the zero matrix with right dimensions, *g* is the gradient, and *S* is in skew functions. By applying superposition theorem to (9) the contribution of each coil carrying current *I* at point *p* in the working space can be calculated as:

(10) $$\vec{W}={\left[\begin{array}{c} \vec{\tau }\\ \vec{F} \end{array}\right]}_{6\times 1}={\left[\begin{array}{cc} S(\vec{m}) & O\\ O & h(\vec{m}) \end{array}\right]}_{6\times 8}{\left[\begin{array}{c} \vec{B(p)}\\ G(p) \end{array}\right]}_{8\times 8}\times I_{8\times 1}$$

if

$${\left[\begin{array}{cc} S(\vec{m}) & O\\ O & h(\vec{m}) \end{array}\right]}_{6\times 8}{\left[\begin{array}{c} \vec{B(p)}\\ G(p) \end{array}\right]}_{8\times 8}=A{\left(m,p\right)}_{6\times 8}$$

where *A* is called the actuation matrix then the amount of current flowing through each coil is:

(11) $$I=A^{†}\times W$$

where † is the pseudo inverse.

### 2.3. Deep Learning

**Figure 4:**
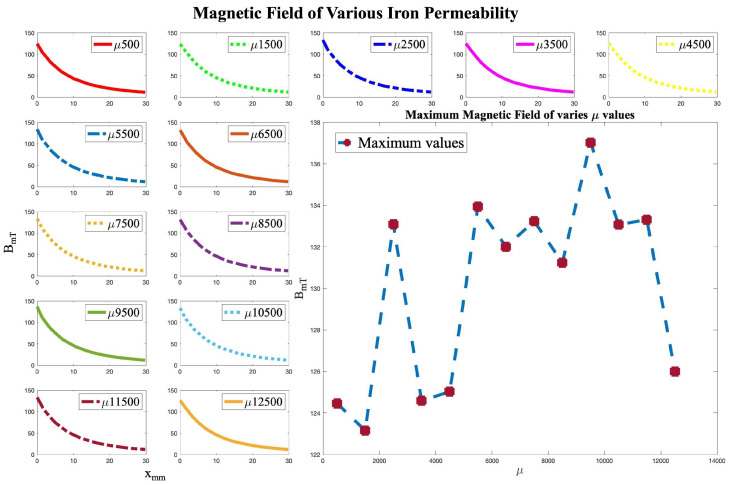
Simulated magnetic field for various magnetic permeability.

**Figure 5:**
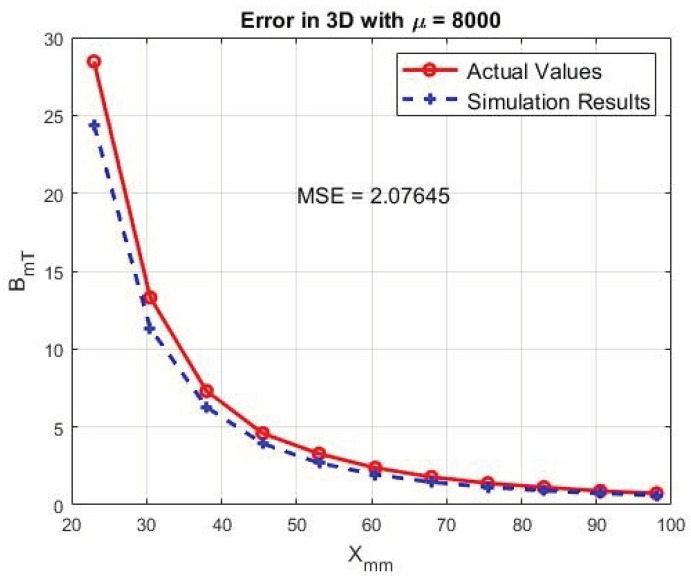
Actual and simulated magnetic field.

As previously mentioned, FEM is used to predict the magnetic field at each point for experimental purposes. However, FEM models cannot capture the manufacturing error caused during the coil winding process. Coil winding is a delicate process and is consistently associated with unwanted gaps, reducing the number of designed turns in the actual coil. In addition, pure iron becomes deformed easily during core manufacturing and changes its dimensions after the annealing process; therefore, even a perfect model cannot represent the actual coil properties and model magnetic field values. In addition to the mentioned barriers, FEM itself is associated with an approximation error that is a part of this approach. Figure 4 indicates the insensibility of the design to permeability change of the iron core. Moreover, Figure 5 illustrates the difference between actual and calculated values of the magnetic field on the axial axis of a coil, with an average error of 17%.

In order to enhance the modeling of the magnetic field and consider human error, deep learning has been used as an alternative to approximate the magnetic field values. Deep learning eliminates the need for collecting too many data points and can be developed with few values inside the region of the interest. Artificial Neural Network (ANN) and a hybrid model, Artificial Neural Network with Simulated Annealing (ANN/SA) algorithm, have been implemented in this study.

#### 2.3.1. ANN

**Figure 6:**
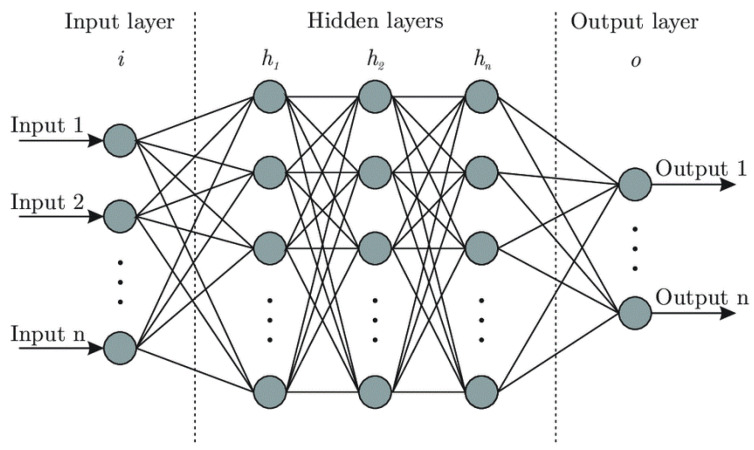
ANN structure.

The artificial neural network is a deep learning approach, a subfield of machine learning, where the structure of the human brain inspires the foundation of the algorithm [31]. The ANN algorithm uses training data to recognize the patterns and predict the outputs for a new set of similar data [32]. The training data is fed to the network as an input layer; then, it moves along channels through hidden layers, which are the core processing units of the network, to the output layer. Figure 6 indicates the general structure of a network. Each channel is assigned to a number known as weight, which scales the neuron from the previous layer and feeds it to the neuron in the next layer. All the channels toward a particular neuron will be added together; then, another value known as the bias or interception point is added to this equation. Finally, an activation function will be applied to the neuron before it leaves toward the next layer through its channels. The weight adjustment process will be done through backpropagation, mainly using the gradient descent approach [33].

**Figure 7:**
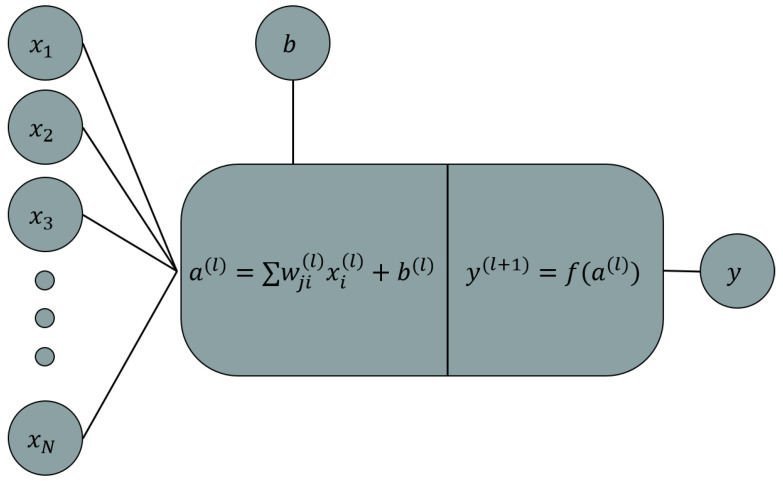
Neuron value calculation.

For most regression problems, the outer layer is only a summation of the last layer’s neuron values multiplied by their associated weights plus the interception point without any activation matrix. Figure 7 illustrates the scaling, bias (*b*), and activation function (*f*) over one neuron only; this process is known as forward propagation [34]. The weight associated with the channel that connects the *j*th neuron of the layer *l* to the *i*th element of the next layer will be shown as $\omega_{ji}^{\left(l\right)}$; the first number in the subscript is the neuron that the channel leaves from, and the second one is where it lands; the superscript also refers to the layer that the channel roots from, for example, the $\omega_{52}^{\left(3\right)}$, which connects the fifth neuron of the third layer to the second neuron of the fourth layer.

(12) $${\left[\begin{array}{c} y_{1}^{\left(l+1\right)}\\ y_{2}^{\left(l+1\right)}\\ \ddots \\ y_{n}^{\left(l+1\right)} \end{array}\right]}_{n\times 1}=f\left({\left[\begin{array}{c} a_{1}^{\left(l+1\right)}\\ a_{2}^{\left(l+1\right)}\\ \ddots \\ a_{n}^{\left(l+1\right)} \end{array}\right]}_{n\times 1}\right)={\left[\begin{array}{cccc} \omega_{11}^{\left(l\right)} & \omega_{21}^{\left(l\right)} & \cdots & \omega_{k1}^{\left(l\right)}\\ \omega_{12}^{\left(l\right)} & \omega_{22}^{\left(l\right)} & \cdots & \omega_{k2}^{\left(l\right)}\\ \vdots & \vdots & \ddots & \vdots \\ \omega_{1n}^{\left(l\right)} & \omega_{2n}^{\left(l\right)} & \cdots & \omega_{kn}^{\left(l\right)} \end{array}\right]}_{n\times k}{\left[\begin{array}{c} x_{1}^{\left(l\right)}\\ x_{2}^{\left(l\right)}\\ \ddots \\ x_{k}^{\left(l\right)} \end{array}\right]}_{k\times 1}+{\left[\begin{array}{c} b_{1}^{\left(l\right)}\\ b_{2}^{\left(l\right)}\\ \ddots \\ b_{n}^{\left(l\right)} \end{array}\right]}_{n\times 1}$$

where *n* and *k* are the number of neurons at the l+1 and the *l* layers, respectively. The weight matrix can also be shown as $\omega^{l}$.

#### 2.3.2. ANN/SA

The ANN algorithm’s performance and convergence are heavily dependent on the initial weight values; therefore, it is beneficial to develop a method that can produce initial weights and biases that increase the probability of reaching the global minimum and increase the learning pace of the network.

**Figure 8:**
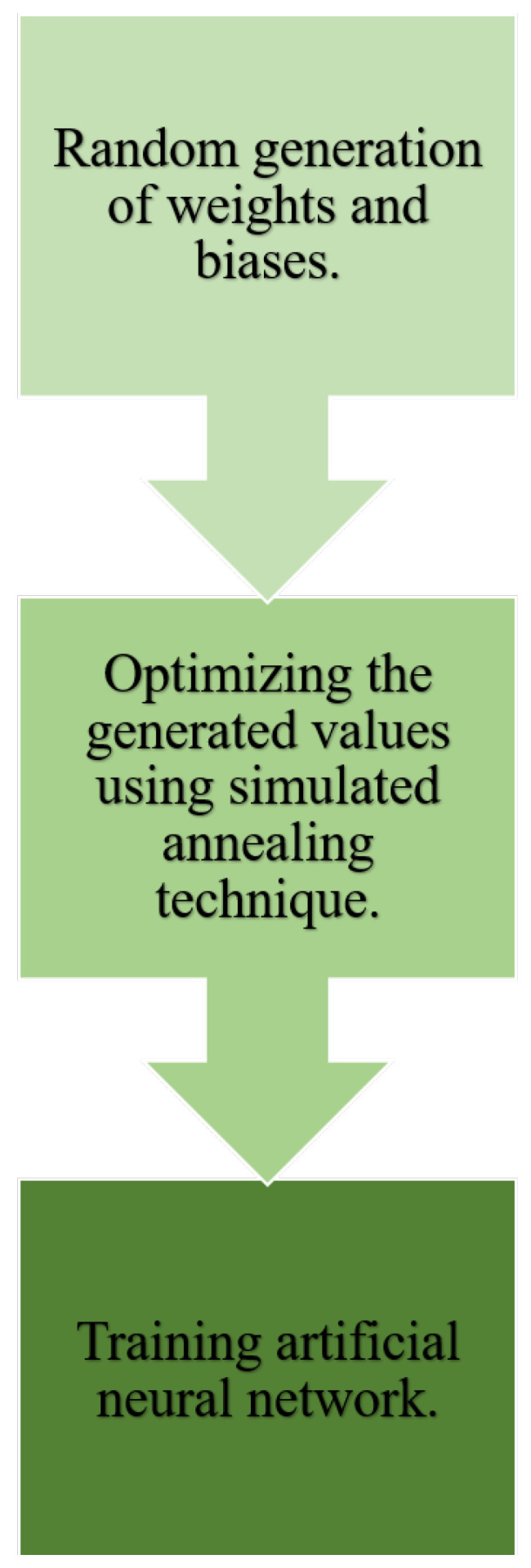
ANN/SA process.

ANN/SA optimizes the randomly generated initial weights and biases values; once the best initial values are selected, the network training process starts. The thermomechanical annealing process inspired the optimization of random values [35]. The metal is heated to a higher temperature and cooled down, resulting in a variation in the metal’s atomic structure and material properties. The atomic structure and the temperature of the metal will be related together, and if the temperature drops slowly, it can be related to the energy change of the metal [36]. Figure 8 illustrates the explained process.

This method was used to optimize nonlinear functions involving multiple local minimums, and it uses the Metropolis algorithm to simulate the annealing process [37]. Since it is not a greedy process, the probability of reaching a local minimum is considerably low. During the training process, the SA will adjust the weights randomly, considering the algorithm’s temperature change and evaluating the network’s accuracy; once the accuracy has been calculated, the Metropolis algorithm decides whether the created solution is acceptable—the probability of acceptance $P_{a}$ is:

(13) $$P_{a}\left(\Delta E,y\right)=\left\{\begin{array}{cc} \exp-\frac{k\Delta E}{y} & \Delta E>0\\ 1 & \Delta E\leq 0 \end{array}\right.$$

where ΔE is the error between the new solution and current solution, *y* is the current temperature, and *k* is the acceptance constant, based on the range of weights, biases, and inputs. As (13) indicates, the algorithm will frequently be accepting new results at high temperatures, however becomes more selective at the lower ones. The cooling schedule can be exponential, linear, and temperature cycling. In order to calculate the temperature at each state using the temperature cycling method, the following equations should be implemented [38].

This method first takes a series of n∈[1,N] scenarios for temperature:

(14) x[n+1]=ρx[n]

where the total number of temperatures is *N*, the starting temperature is x[1], the final temperature is x[N], and cooling constant is ρ presented by:

(15) $$\rho =e^{\log(\frac{\left(N-1)x[N\right]}{x[1]})}.$$

If the number of cycles before each optimization is expressed as *M* and the temperature assigned to the simulated annealing indicated by y[n], the schedule of the cooling process can be modeled as:

(16) $$y\left[n\right]=\sum_{m=0}^{M-1}x\left[n-mN\right]$$

ultimately, the weight preparation of the layer *l* for the temperature can be formulated as:

(17) $$\omega_{ij}^{l}\left[n+1\right]=\gamma \left(1-\lambda \right)\omega_{ij}^{l}\left[n\right]+\lambda u\left[n\right]-0.5\lambda$$

where *u* is a random variable that is uniformly distributed and takes values between [0,1), γ is a value equal to 20 or 30, and λ is the perturbation ratio:

(18) $$\lambda =\frac{y[n]}{x[1]}.$$

#### 2.3.3. Validation

In order to evaluate the performance of each developed algorithm and select the best approximation, Root-Mean-Square-Error (RMSE) and R-Squared $\left(R^{2}\right)$ are utilized.

(19) $$RMSE=\sqrt{\frac{1}{N}\sum_{i=1}^{N}(y_{i}-{\hat{y}}_{i}{)}^{2}}$$

(20) $$R^{2}=1-\frac{\sum_{i=1}^{N}(y_{i}-{\hat{y}}_{i}{)}^{2}}{\sum_{i=1}^{N}(y_{i}-{\bar{y}}_{i}{)}^{2}}$$

where $y_{i}$, $\hat{y_{i}}$, $\bar{y_{i}}$, and *N* are the actual, predicted, mean of actual set values, and the number of samples, respectively. Once each model had been optimized, the final algorithm will be additionally evaluated using the values mentioned in Table 1 to check its predictability [39,40].

## 3. Results & Discussion

### 3.1. Designed Coil

**Figure 9:**
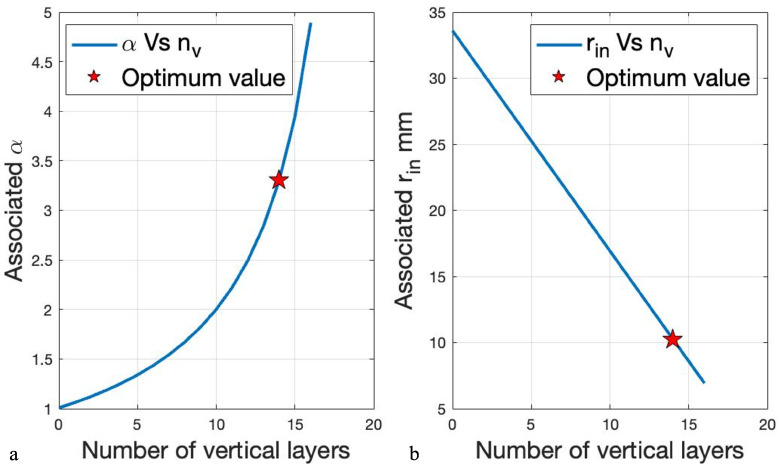
(**a**) Number of layers from the inner radius toward outer radius for various α. (**b**) Inner radius calculation based on the number of layers for different α values.

The coil is wound using an AWG 14 wire with a diameter of 1.73228 mm with its isolation. The α value for the various number of vertical layers has been investigated considering the diameter of the wire and the geometric restriction of 67.69 mm of the coil’s outer diameter. As indicated in Figure 9a, the optimum value of α=3.3019 occurs at 14 layers. Moreover, as Figure 9b illustrates, the calculation for optimum feasible alpha value starts when inner and outer radiuses are equal, meaning that the thickness is zero, then it continues by adding one layer at each step and calculates the alpha value accordingly. Figure 9 indicates that the optimum α value is associated with a 10.25-mm inner radius.

**Figure 10:**
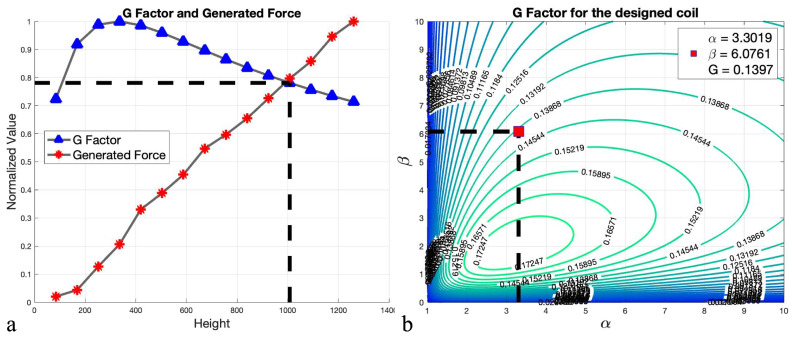
(**a**) Normalized values of different scenarios. (**b**) Optimum coil parameters.

The coil’s inner radius selection leads to β optimization. As indicated in Table 2, by varying the length of the coil and modeling each scenario on Ansys Maxwell 2020 the magnetic force had been calculated. According to the Figure 10, which is the illustration of the normalized force and Fabry factor indicated in Table 2, a length of 124 mm indicates a coil that provides almost as much force with less power consumption than the next scenario. Therefore, the optimum coil is a cylindrical iron-core coil with 3.3019,6.0761, and 0.140 for α, β, and *G* values, respectively.

### 3.2. Data Collection

**Figure 11:**
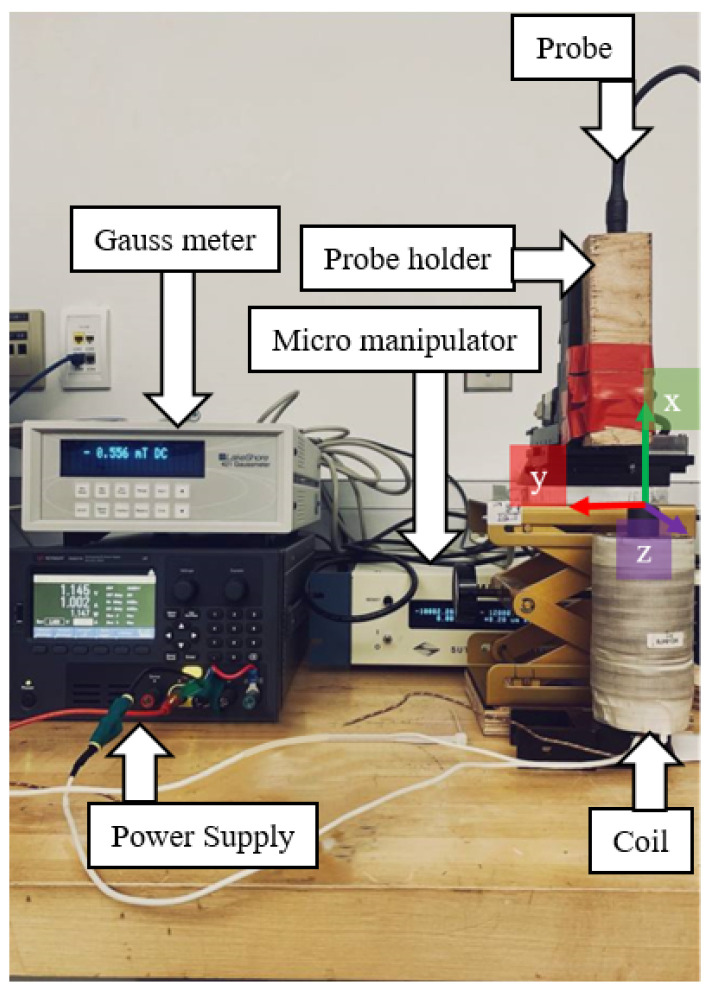
Magnetic field values collection setup.

As indicated in Figure 11, the magnetic field had been measured using a Gauss meter over a 7×7×6 grid, where y,z∈ [−10 mm, 10 mm] and x∈ [52 mm, 72 mm]. A total of 294 points for a coil running at one amp were collected for each magnetic field component. The input data is the location of the point of interest above the coil, and the target or label is the magnetic field components, $B_{x}$, $B_{y}$, and $B_{z}$.

**Figure 12:**
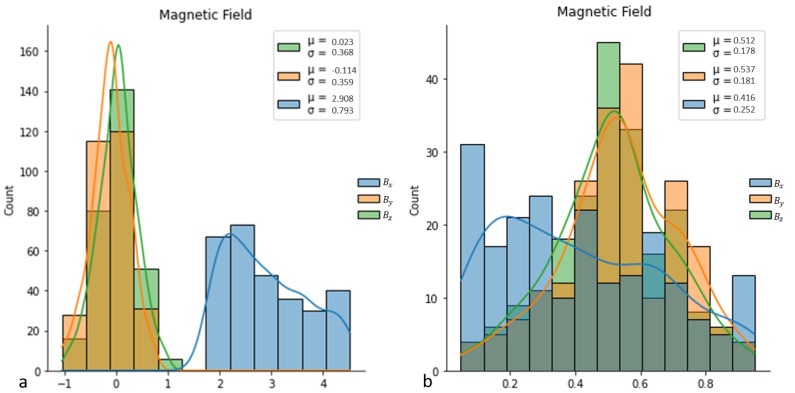
(**a**) Original data. (**b**) Normalized data.

In order to guarantee the convergence of the algorithms and reach the global minimum, the collected data have been normalized using the upper-lower approach between 0.05 and 0.95; Figure 12 shows the effect of normalization on targets.

### 3.3. Algorithm Development

Each target has its own algorithm developed separately for each method. However, to ensure that the algorithms receive the same samples, the data set associated with targets had been randomly shuffled and split into three categories: train, test, and validation sets. The train set consists 70 percent of the initial data, with 206 samples, whereas the test and validation sets are filled with only 15 percent, with 44 samples each. Each model is developed using the train set, where its hyperparameters are tuned using the test set. Finally, the algorithm’s performance is measured on validation as unseen data to ensure the accuracy of the model.

#### 3.3.1. ANN

**Figure 13:**
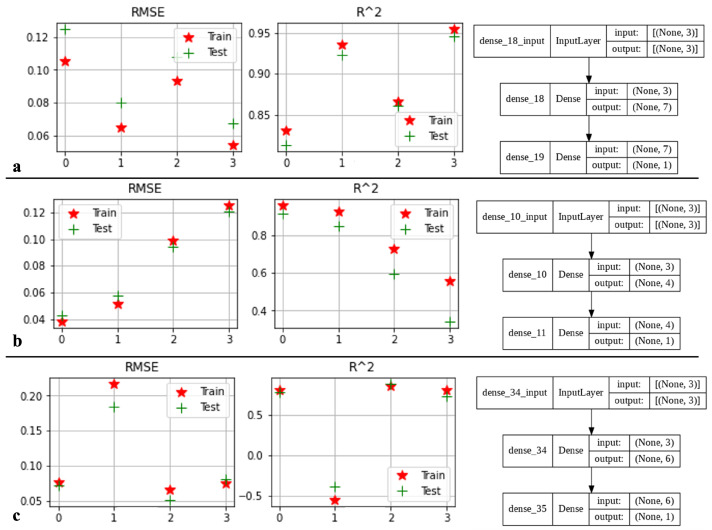
Different scenarios using ANN for (**a**) $B_{x}$, (**b**) $B_{y}$, and (**c**) $B_{z}$.

**Figure 14:**
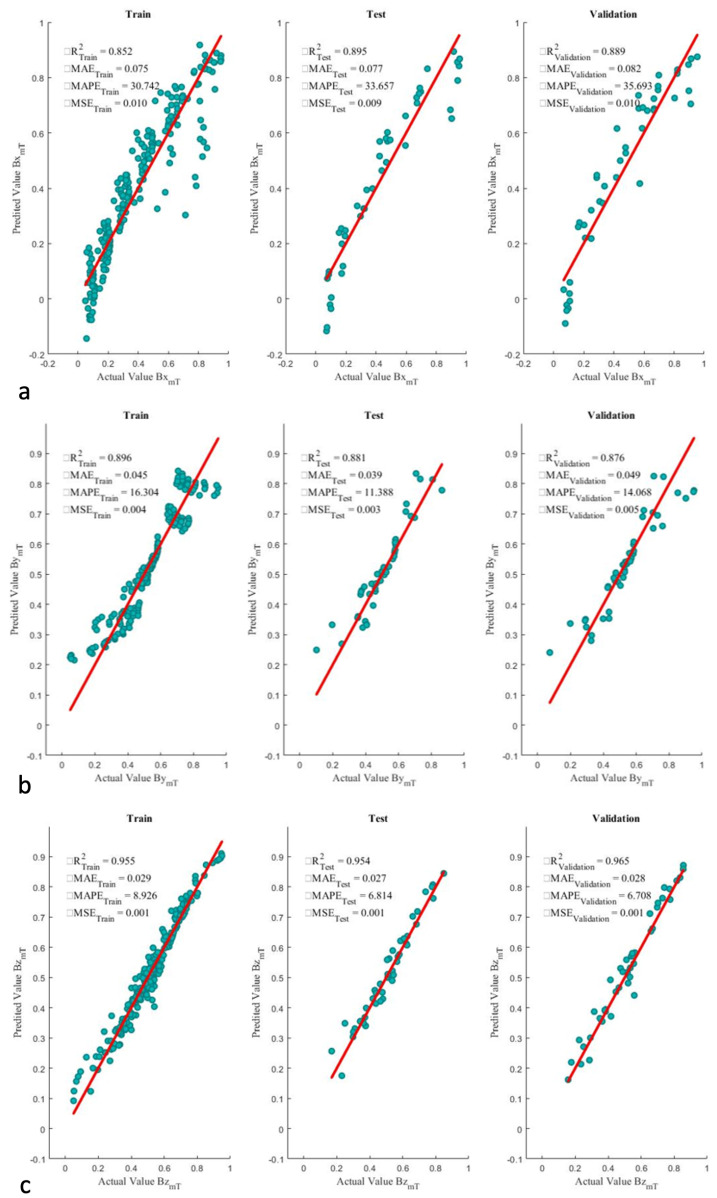
Scatter plot of optimized ANN algorithms for (**a**) $B_{x}$, (**b**) $B_{y}$, and (**c**) $B_{z}$.

The network has been developed using one hidden layer consisting of four to seven neurons. The Adam optimizer, stochastic gradient descent method, a learning rate of 0.001, and batch size of 64 was chosen. The hidden layer is formed by activating a relu function, and the output layer is the linear combination of the neurons and biases times to associated weights of the hidden layer. Figure 13 indicates the number of neuron selection process based on $R^{2}$ and RMSE for each target. The highest $R^{2}$ and lowest RMSE are associated with seven, four, and six neurons for $B_{x}$, $B_{y}$, and $B_{z}$, respectively. Figure 14 shows the implementation of the developed algorithm on the train, test, and validation sets. $B_{z}$ reached $R^{2}>0.950$ on all the sets; whereas, $B_{x}$ and $B_{y}$ reached as far as $R^{2}=0.889$ and $R^{2}=0.876$.

#### 3.3.2. ANN/SA

**Figure 15:**
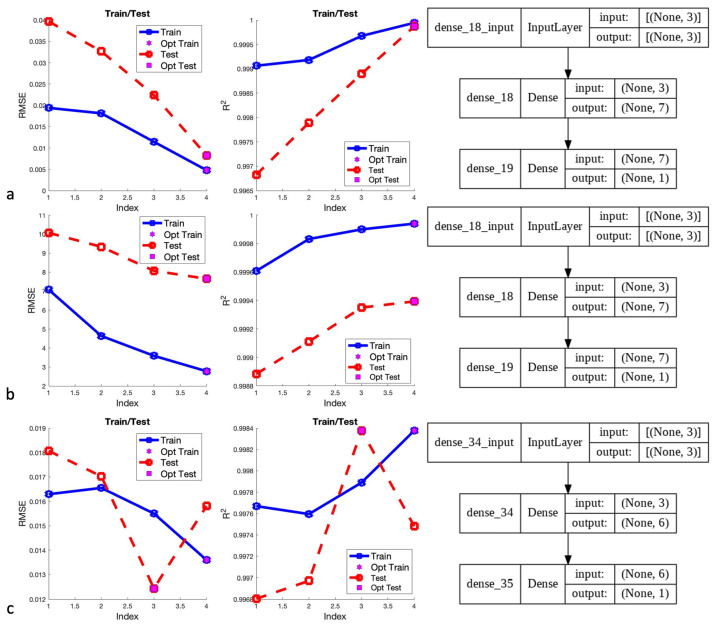
Different scenarios using ANN/SA for (**a**) $B_{x}$, (**b**) $B_{y}$, and (**c**) $B_{z}$.

The network was created using similar data and structure as ANN. By using temperature cycling for the cooling cycle with initial and final temperatures of 15 and 0.015, the data set associated with each magnetic field component was trained and tested in NeuralLab v.3.1 software. Models indicated that the Levenberg–Marquardt algorithm had worked better in all cases than the gradient descent. According to Figure 15, $B_{x}$ and $B_{y}$ components have performed better with seven neurons than other scenarios. Moreover, Figure 15 indicates that among four scenarios, a structure with seven neurons $B_{z}$ is the optimal structure on the training data; however, six neurons are better than others on the test set. Since performance on the test set is more important than training and the six-neuron structure is the second best on the training set, the optimum structure will be six neurons.

**Figure 16:**
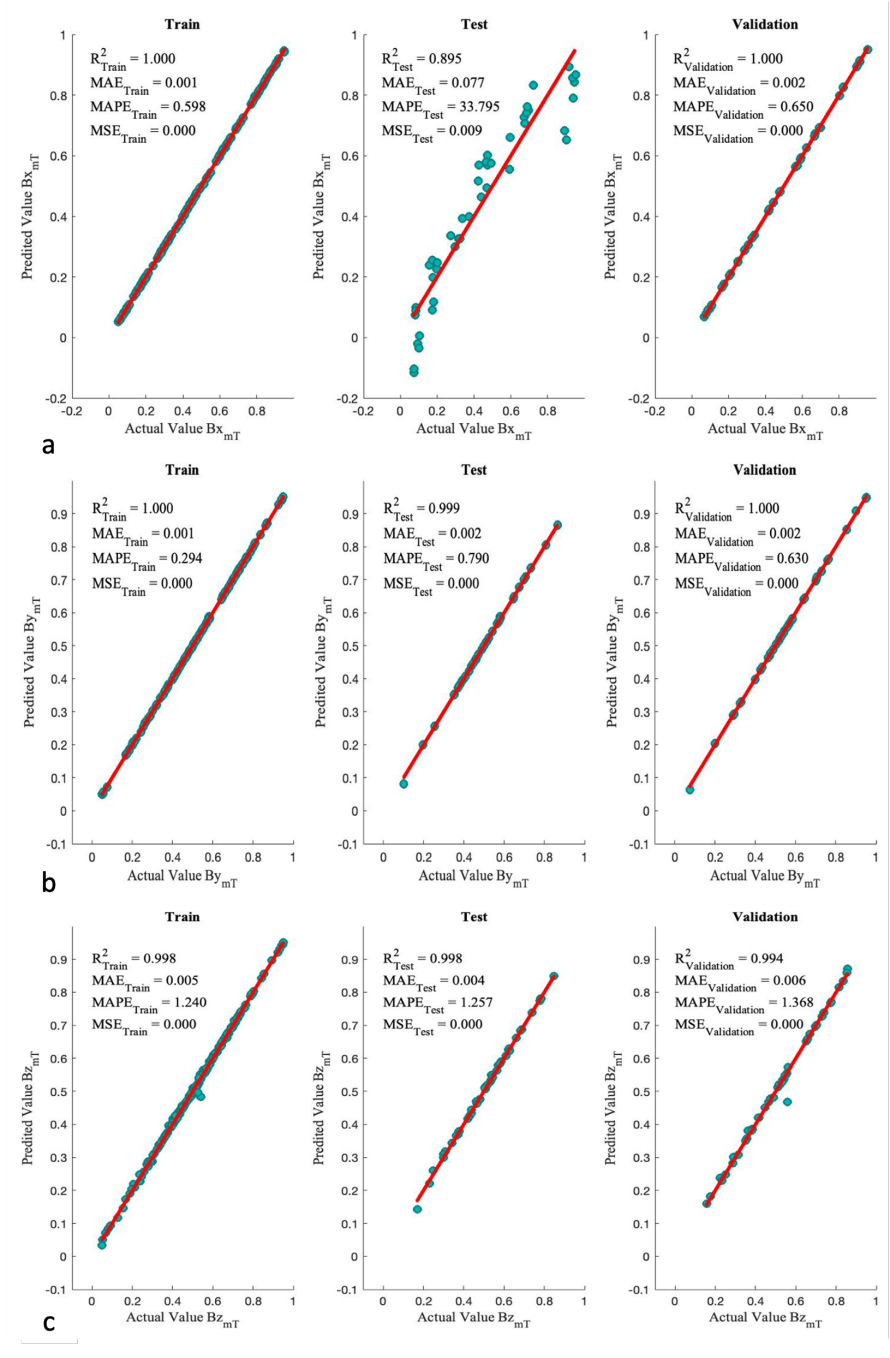
Scatter plot of optimized ANN/SA algorithms for (**a**) $B_{x}$, (**b**) $B_{y}$, and (**c**) $B_{z}$.

Figure 16 illustrates the scatter plots with associated $R^{2}$ and error values for optimal structures. The performance of this approach shows a significant improvement compared to the ANN approach. The scatter plots closely follow the red line and has a minimal residual. However, the test performance of the Bx component is very similar to the ANN performance.

#### 3.3.3. Extra Validation

According to Figure 14 and Figure 16, the ANN/SA is a better approach to approximate the magnetic field than the other one. Therefore, the predictability of this algorithm on the validation set has been checked as the final performance measurement. As Table 3 indicates, all the variables are in the expected range, and the regression models can be used for manipulation purposes.

**Figure 17:**
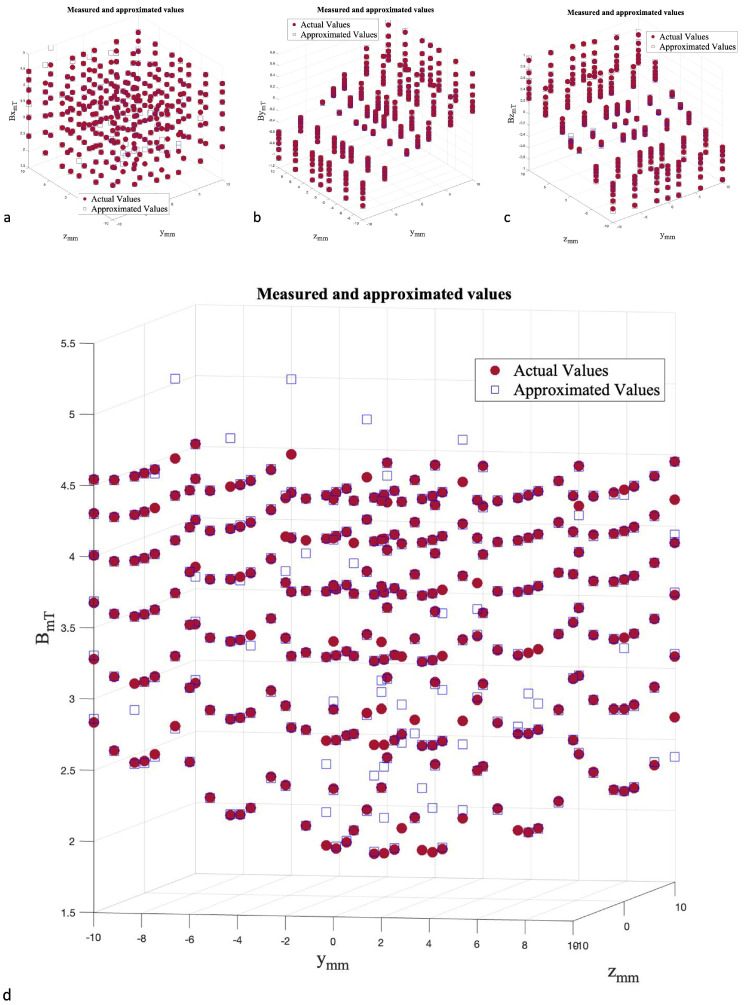
3D Scatter plots of optimized ANN/SA algorithms for (**a**) $B_{x}$, (**b**) $B_{y}$, (**c**) $B_{z}$, and (**d**) *B*.

Figure 17 indicates the field values over the 7×7×6 grid, where y,z∈ [−10 mm, 10 mm] and x∈ [52 mm, 72 mm]. As can be seen, the actual values and approximated values are close to each other and the mean error percentage is 0.0633%.

### 3.4. Motion

**Figure 18:**
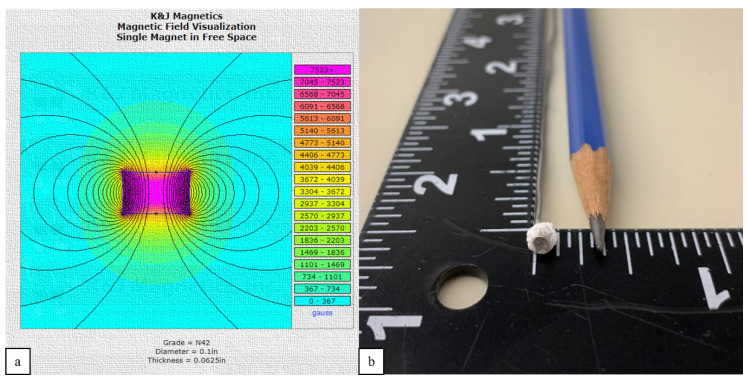
The agent’s dimensions (**a**) and properties from the manufacturer [41] (**b**) with the attached flexible part in inch.

**Figure 19:**
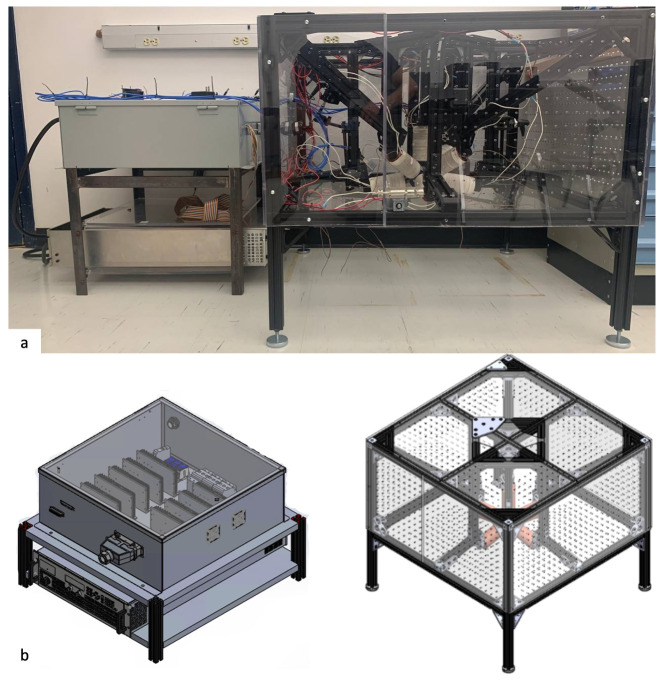
(**a**) Fabricated frame and (**b**) designed frame.

The developed algorithm has indicated reliable results and can be utilized for manipulation purposes. Each designed coil also can produce a maximum of 83.43 mH magnetic field at a maximum current of 10 amp, which is enough to manipulate at a milli/micro scale. Figure 18 is a representation of a magnet attached to a flexible road acting as the robot. This disk magnet has a dipole moment of 0.0084 Am^2^ with a diameter and thickness of 0.1 and 0.0625 inches. The agent will be navigated only in one direction toward a laser sensor which provides real-time position feedback of the agent. Since there is no other information, such as the agent’s orientation, provided by the laser sensor, the system can become unstable quickly. To overcome the rotational instabilities, the attached flexible part to the agent was designed to be heavier than its normal weight. Figure 19 indicates the fabricated system utilized to run the experiment.

**Figure 20:**
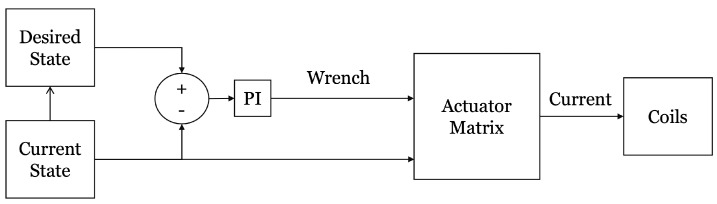
The actuation logic.

**Figure 21:**
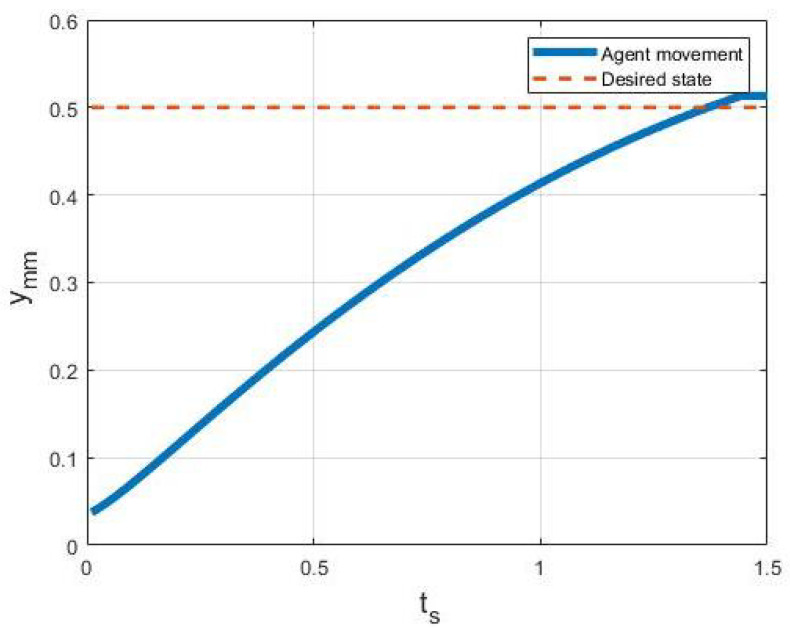
Robot movement toward 0.5 mm.

The provided position by the laser sensor will be used to calculate the amount of required current at each coil utilizing (11). The actuation logic, indicated in Figure 20, has resulted in the movement of the robot with an acceleration rate of 0.228 ${\mathrm{mm}/s}^{2}$ toward a 0.5-mm point resulting in a movement accuracy of 13 μm. In this logic, the current state of the robot is collected from the laser sensor and compared to the desired point. The motion continues until the difference between the desired and current points is considerably small, meaning that the associated wrench cannot overcome the friction force. Figure 21 illustrates the movement of the robot with respect to time.

## 4. Conclusions

This work demonstrated a magnetic manipulator’s design and implementation process with eight coils. The integration of the Fabry factor and force strength has led to an optimal coil with an inner radius, outer radius, and height of 20.5, 67.69, and 124.56 mm, respectively. Due to the error associated with FEM, 17% in this study, the magnetic field produced by this coil at one amp has been collected over a 7×7×6 grid and approximated using deep learning techniques, ANN and ANN/SA. Although both models showed an acceptable performance, the ANN/SA showed more reliable performance values with an average of RSME=0.0153 and $R^{2}=0.9871$, 76.61% less and 9.66% more than the ANN model, respectively. Utterly, the developed algorithm was used to approximate the magnetic field and navigate an agent from almost the center of the working space toward a half-millimeter away from it along a laser sensor direction. The manipulation had been implemented successfully with 13 μm of accuracy.

## Figures and Tables

**Table 1:** Extra evaluation parameters.

Variable	Equation	Criteria
Mean Absolute Error (MAE)	MAE= $\frac{\sum_{i=1}^{N}\left|(y_{i}-{\hat{y}}_{i})\right|}{N}$	As low as possible
Mean Absolute Percentage Error (MAPE)	MAPE= $\frac{100}{N}\sum_{i=1}^{N}|\frac{y_{i}-{\hat{y}}_{i})}{y_{i}}|$	As low as possible
Slope regression line *k*	*k* = $\frac{\sum_{i=1}^{N}\left(y_{i}\times \hat{y_{i}}\right)}{y_{i}^{2}}$	0.85<k<1.15
Slope regression line $k^{{}^{\prime }}$	$k^{{}^{\prime }}=\frac{\sum_{i=1}^{N}\left(y_{i}\times \hat{y_{i}}\right)}{{\hat{y_{i}}}^{2}}$	$0.85<k^{{}^{\prime }}<1.15$
Squared correlation of actual vs predicted $\left(Ro^{2}\right)$	$\left(Ro^{2}\right)$ = $1-\frac{\sum_{i=1}^{N}(\hat{y_{i}}-{y^{o}}_{i}{)}^{2}}{\sum_{i=1}^{N}(\hat{y_{i}}-\bar{\hat{y_{i}}}{)}^{2}}$ ${y_{i}}^{o}=k\times \hat{y_{i}}$	Close to 1
Squared correlation of predicted vs actual ${\left(Ro^{{}^{\prime }}\right)}^{2}$	${\left(Ro^{{}^{\prime }}\right)}^{2}$ = $1-\frac{\sum_{i=1}^{N}(y_{i}-{\hat{y_{i}}}^{o}{)}^{2}}{\sum_{i=1}^{N}(y_{i}-\bar{y_{i}}{)}^{2}}$ ${\hat{y_{i}}}^{o}=k^{{}^{\prime }}\times y_{i}$	Close to 1
Predictability of model $R_{m}$	$R_{m}$ = $R^{2}\times \left(1-\sqrt{|R^{2}-R_{o}^{2}|}\right)$	$R_{m}>0.5$
Performance index *m*	*m* = $\frac{R^{2}-Ro^{2}}{R^{2}}$	|m|<0.1
Performance index *n*	*n* = $\frac{R^{2}-{Ro^{{}^{\prime }}}^{2}}{R^{2}}$	|n|<0.1

**Table 2:** Different coils with their generated force.

Length_mm_	β	G	Force_mT_
10.38	0.5063	0.129	0.013
20.76	1.0127	0.164	0.027
31.14	1.5190	0.177	0.080
41.52	2.0254	0.179	0.129
51.90	2.5317	0.176	0.207
62.28	3.0380	0.172	0.243
72.66	3.5444	0.166	0.284
83.04	4.0507	0.160	0.342
93.42	4.5571	0.155	0.373
103.80	5.0634	0.149	0.409
114.18	5.5698	0.144	0.454
124.56	6.0761	0.140	0.497
134.94	6.5824	0.135	0.536
145.32	7.0888	0.131	0.591
155.70	7.5951	0.128	0.625

**Table 3:** Extra evaluations for ANN/SA.

Variable	$B_{x}$	$B_{y}$	$B_{z}$	Criteria
*k*	0.9995	0.9988	0.9985	0.85<k<1.15
$k^{{}^{\prime }}$	1.0005	1.0012	1.0007	$0.85<k^{{}^{\prime }}<1.15$
$\left(Ro^{2}\right)$	1.0000	1.0000	1.0000	Close to 1
${\left(Ro^{{}^{\prime }}\right)}^{2}$	1.0000	1.0000	1.0000	Close to 1
$R_{m}$	0.9912	0.9867	0.9161	$R_{m}>0.5$
*m*	−0.0001	$-0.1727\times 10^{-3}$	−0.0062	|m|<0.1
*n*	−0.0001	$-0.1727\times 10^{-3}$	−0.0062	|n|<0.1

## Data Availability

You can request data using the Maglev Laboratory at University of Waterloo.

## Abbreviations

The following abbreviations are used in this manuscript:

FEM	Finite Element Method
AI	Artificial Intelligence
ANN	Artificial Neural Network
ANN/SA	Artificial Neural Network with Simulated Annealing
DOF	Degrees Of Freedom
CGCI	Catheter Guidance Control and Imaging
